# Association between endothelin-1, nitric oxide, and Gensini score in chronic coronary syndrome

**DOI:** 10.1186/s12872-023-03625-w

**Published:** 2023-12-08

**Authors:** Yujin Wang, Yuqin Wang, Tiaoxia Liu, Yifan Qin, Futian Tang, Xiaowei Zhang, Yongnan Li

**Affiliations:** 1 Lanzhou University Second College of Clinical Medicine, Lanzhou, China; 2 Department of Cardiology, Lanzhou University Second Hospital, #82 Cuiyingmen, Lanzhou, 730030 China; 3 School of Nursing, Lanzhou University, Lanzhou, Gansu China

**Keywords:** Gensini score, Endothelin-1, Nitric oxide, Chronic coronary syndrome

## Abstract

**Background:**

Chronic coronary syndrome (CCS) is a major public health burden; its pathogenesis involves atherosclerosis and endothelial dysfunction. Endothelin-1 (ET-1) and nitric oxide (NO) are vasoactive substances synthesized by endothelial cells that play a crucial role in CCS development. The Gensini score (GS) is used for evaluating CCS severity based on lumen segment changes, stenosis degree, and coronary stenosis site.

**Methods:**

This prospective study included 71 patients with CCS; we evaluated the relationships between GS and ET-1 and NO serum levels were evaluated in these patients. The GS was calculated for all patients. Serum ET-1 & NO levels among other laboratory parameters were measured.

**Results:**

The high GS group had higher ET-1 and relatively NO expressions in the than the low GS group. GS was positively correlated with ET-1 and negatively correlated with NO, T4, and TSH levels. The results of the multiple linear regression analysis showed that ET-1 had the most significant effect on GS.

**Conclusions:**

We found a strong association between ET-1, NO, and CCS severity. A combination of ET-1, NO, and GS is an essential predictor of CCS disease severity.

## Introduction

Worldwide, cardiovascular disease (CVD) is the leading cause of death [1] and chronic coronary syndrome (CCS) is a significant public health burden [2]. CCS, the term for the clinical symptom of coronary atherosclerosis-induced coronary lumen stenosis, weakened vasodilation, ischemia, and hypoxia [3], is induced by various factors, including atherosclerosis and vascular endothelial cell dysfunction [4, 5]. Vascular endothelial cells cover the internal blood vessel walls as a single layer [4]. Endothelial cells not only create a permeable barrier but also have endocrine and paracrine functions wherein they express and secrete multiple biologically active mediators that regulate vascular tone, thrombosis, smooth muscle cell growth, immune response, extracellular matrix, and inflammatory reaction [5]. Endothelin-1 (ET-1) and nitric oxide (NO), which are vasoactive substances synthesized by endothelial cells, have enhancing and protective effects on CCS, respectively [6–8]. ET-1, a vascular contraction factor, binds to its receptor to exert its biological effects. ET-1 was reported to predict adverse outcomes, including mortality in patients after acute myocardial infarction and congestive heart failure diagnosis [9]. A 20-year prospective study showed that high ET-1 plasma levels improve the preclinical atherosclerosis predictive value for future cerebrovascular and cardiovascular events [10]. Additionally, it is known that serum ET-1 levels positively correlate with waist and hip circumferences in CCS patients [11], and low plasma NO level is a significant risk factor for CCS development and severity [12] and high NO bioactivity can treat CCS [6]. The endothelium regulates vascular tone by balancing production of vasodilators (NO). A family history of premature atherosclerotic disease are all associated with an attenuation/loss of endothelium-dependent vasodilation [13]. NO and ET-1 are natural counterparts in vascular function, and an imbalance between these two mediators is a characteristic of endothelial dysfunction and may determine the onset and degree of certain cardiovascular diseases [14]. Gensini score (GS) was introduced in 1983 [15] and is used for determining CCS severity [16] based on changes in the lumen segment, stenosis degree, and coronary stenosis site [17, 18]. Research has confirmed the crucial value of GS in assessing CCS condition [18], and it is an independent predictor of long-term adverse outcomes in patients with CCS who have undergone percutaneous coronary intervention (PCI) [19]. A retrospective study found the GS of patients with CCS to be significantly different from that of healthy individuals; furthermore, statistical analysis showed a correlation between GS and patient prognosis [18]. Nevertheless, the relationship between GS and ET-1 and NO serum levels in patients with CCS needs elucidation. We aimed to investigate the significance and association of GS and ET-1 and NO serum levels in patients with CCS to elucidate potential CCS diagnosis and prognosis predictors.

## Materials and methods

### Study population

This prospective study included 71 patients with CCS at the Lanzhou University Second Hospital between October 2020 and May 2021. The study was approved by the Ethics Committee of the Lanzhou University Second Hospital (2021 A-022). All patients provided written informed consent.

### Research methods

After hospital admission, all patients completed the relevant baseline and psychological scale surveys, physical examinations, and laboratory tests. Coronary angiography (CAG) was done 12–24 h postoperatively. The inclusion criteria were: (1) patients who met the American Guidelines for the Diagnosis and Treatment of Coronary Heart Disease diagnostic criteria for CCS [20] and showed obvious symptoms; (2) conscious patients who had no communication difficulties; and (3) complete medical records. The exclusion criteria were: (1) the presence of mental disorders or mental retardation; (2) the presence of other cardiovascular diseases, including congenital heart disease, cardiomyopathy, etc.; (3) a history of heart transplantation; (4) the presence of other severe medical conditions, including heart failure, respiratory failure, etc.; (5) presence of systemic immune diseases or infections; and (6) pregnant or breastfeeding patients; (7) Age < 18 years; (8) presence of peripheral artery disease.

### Clinical data collection

Baseline patients data, including age, gender, ethnicity, smoking history, drinking history, body mass index (BMI), heart rate (HR), percutaneous coronary intervention (PCI), systolic blood pressure (SBP), diastolic blood pressure (DBP), SBP standard deviation (SBPSD), DBP standard deviation (DBPSD), SBP variation (SBPV), DBP variation (DBPV), forced vital capacity (FVC), forced expiratory volume in the first second (FEV1), maximum ventilation volume (MVV), 6-minute walk test (6MWT), and anxiety scale scoring, were collected from an electronic medical record system. Hematological parameters, including hemoglobin (HB), C-reaction protein (CRP), creatinine (CR), bloodglucose (Glu), kinase isoenzyme (CK-MB), cardiac troponin I (TnI), prothrombin time (PT), prothrombin activity (PT%), thrombin time (TT), activated partial thromboplastin time (APTT), prothrombin time ratio (PT-R), international normalized ratio (INR), plasma fibrinogen (FIB), antithrombin III (AT III), and fibrinogen degradation product (FDP) were also measured. The serum total cholesterol (TC), triglyceride (TG), low-density lipoprotein cholesterol (LDLC), high-density lipoprotein cholesterol (HDLC), triiodothyronine (T3), thyroxine (T4), free triiodothyronine (FT3), free tetraiodothyronine (FT4), thyroid stimulating hormone (TSH) levels were also measured.

### Determination of ET-1 and NO

Serum NO levels were determined using the nitrate reductase method following manufacturer instructions (A013-2-1, Jiancheng, Nanjing) as described previously. The specific steps are as follows. (1) Add the sample and R1, R2 mixed reagents according to the steps, and take a water bath at 37 ℃ for 60 min. (2) Add R3 and R4, extract and let stand at room temperature for 40 min, 3500–4000 rpm, centrifuge for 10 min. (3) Take 0.5 ml of supernatant, add chromogenic agent, let stand for 10 min, 550 nm wavelength, 1 cm optical path, colorimetric analysis. Moreover, blood samples were taken from patients and control subjects at 6–7 am after an overnight fast. After centrifugation at 3000 r/min for 10 min, the supernatant was collected and stored at − 80 °C. Serum levels of ET-1, was measured using enzyme-linked immunosorbent assay (ELISA) kits (H093-1-1, Jiancheng, Nanjing) following the manufacturer’s instructions as described previously.

### Cardiopulmonary function

The cardiopulmonary exercise test is an internationally accepted cardiopulmonary test that measures respiratory and circulatory function levels [21] and can also evaluate functional motor capacity and disease diagnosis and treatment. To ensure patient safety and considering that the patient had not received any therapeutic intervention, the exercise intensity was kept close to daily human activities. The patients undertook the 6MWT and walked as swiftly as possible to increase exercise intensity and evaluate the ability of sub-extreme exercise. The P, R, SPO_2_, SBP, DBP, SBPSD, DBPSD, SBPV, DBPV, FVC, FEV1, and MVV indicators and walking distance were measured during the exercise. FVC is the maximum air volume that can be exhaled in the shortest possible time after maximum inhalation. FEV1, a functional index, indicated clinical lung function and is calculated as the lung gas volume percentage that an individual can forcibly expel during the first second following maximal inhalation. Similarly, MVV is the maximum volume of air an individual can inhale and then exhale in one minute.

### Gensini score assessment

CAG was performed by the same professional intracardiac intervention team for all patients, and its results, from which the GSs were calculated, were interpreted by two deputy chief physicians. GS is evaluated as follows: (1) The coronary artery is divided into 15 segments, and weight coefficients are assigned based on the segments and parts of the coronary artery. The weight coefficient is 1.5 for the proximal segment; 1 for the left anterior descending branch; 1 for the first diagonal branch; and 1 each for the blunt marginal branch, the posterior branch of the left circumflex, the proximal right coronary artery, the middle of the right coronary artery, the distal right coronary artery, and the posterior descending branch. (2) The coronary stenosis degree determines the weight coefficient as follows: 1 for coronary stenosis ≤ 25%, 2 for 25% < coronary stenosis ≤ 50%, 4 for 50% < coronary stenosis ≤ 75%, 8 for 75% < coronary stenosis ≤ 90%, 16 for 90% < coronary stenosis ≤ 99%, and 32 for coronary stenosis = 100%. (3) The final scoring, that is, the sum of the stenosis scores of each branch vessel, is calculated as the weight coefficient of the coronary stenosis degree multiplied by the weight coefficient of each diseased vessel segment.

### Statistical analysis

PASS 15 was used to calculate the sample size, the minimum sample size required for this study was 32 with α = 0.1 and a lower one-tailed test. Statistical Package for the Social Science, version 23 (SPSS Inc., Chicago, IL) was used for statistical analysis. Measurement data were expressed as mean ± standard deviation and between-group comparisons were done using the analysis of variance (ANOVA) test. Enumeration data were expressed as frequency (%), and the chi-square test was used for between-group comparison. Multivariable linear regression analysis was performed to investigate the relationship between the Gensini score and CCS serum vasoactive substances in CCS. All tests were two-tailed, and p < 0.05 was considered statistically significant.

## Result

### Baseline characteristics of different groups

After the application of the inclusion and exclusion criteria, 71 patients were included in this study. The average age of the patients was 59.21 ± 7.75 years, and 47 (66.2%) patients were male. The patients were divided into two groups according to the median GS: a low GS group (n = 36, GS ≤ 3.5) and a high GS group (n = 35, GS > 3.5) (Table 1). There were significant differences between the two groups in terms of the ET-1 (P < 0.05) and NO (P < 0.01) levels. No significant difference was found in the other indicators (Table 1).

**Table 1:** Baseline characteristics of different groups (N = 71)

Characteristic	Low GS (≤ 3.5, n = 36)	High GS (> 3.5, n = 35)	P value
Age (years)	58.64 ± 7.82	59.8 ± 7.74	0.532
Gender			0.121
Male	21 (44.7%)	26 (55.3%)	
Female	15 (62.5%)	9 (37.5%)	
Smoking			0.533
Yes	24 (50%)	24 (50%)	
No	12 (52.2%)	11 (47.8%)	
PCI	12(33.3%)	12(36.4%)	0.792
BMI (kg/m^2^)	24.95 (23.23, 27.63)	24.3 (23.3, 26.4)	0.221
CRP (mg/L)	0.49(0.49, 0.49)	0.49(0.49, 0.49)	0.777
CR (umol/L)	64.94 ± 12.93	69.07 ± 12.00	0.406
h (bpm)	89.03 ± 12.88	88.70 ± 13.88	0.557
HB (g/L)	151.35 ± 13.73	149.88 ± 11.12	0.424
Glu (mmol/L)	0.49(0.49, 0.49)	0.49(0.49, 0.49)	0.013
CK-MB (ng/mL)	0.09(0.09,1.10)	0.09(0.09,1.75)	0.093
TnI (ng/mL)	0.49(0.49, 0.49)	0.49(0.49, 0.49)	0.083
PT (s)	10.8 (10.53, 11.35)	10.8 (10.1, 11.2)	0.287
PT_R	0.98 (0.95, 1.04)	0.98 (0.92, 1.02)	0.292
INR (U)	0.98 (0.96, 1.03)	0.98 (0.92, 1.02)	0.264
APTT (s)	31.25 (28.63, 32.9)	30.0 (27.6, 31.5)	0.218
FIB (g/L)	3.00 ± 0.48	3.17 ± 0.68	0.223
TT (s)	15.7 (14.63, 16.48)	15.3 (14.8, 15.7)	0.216
ATIII (%)	107.5 ± 17.03	106.06 ± 18.06	0.73
FDP (µg/ml)	0.77 (0.40, 1.11)	0.7 (0.43, 1.20)	0.936
TC (mmol/L)	3.795 (3.16, 4.38)	3.34 (2.88, 4.67)	0.633
TG (mmol/L)	1.72 ± 1.06	1.62 ± 0.64	0.637
HDLC (mmol/L)	1.22 ± 0.37	1.18 ± 0.26	0.567
LDLC (mmol/L)	2.38 (1.9, 2.75)	2.10 (1.67, 3.16)	0.717
T3 (nmol/L)	1.65 ± 0.34	1.62 ± 0.29	0.658
T4 (nmol/L)	107.4 (92.35, 126)	97 (88.5, 114.7)	0.182
FT3 (pmol/L)	5.08 (4.65, 5.57)	5.28 (4.80, 5.74)	0.192
FT4 (pmol/L)	15.16 ± 2.11	15.46 ± 2.61	0.594
TSH (UIU/ML)	2.64 (1.54, 4.57)	2.12 (1.10, 3.29)	0.169
SBP (mmHg)	93 (87.25, 97.75)	90 (85, 103)	0.877
DBP (mmHg)	64.44 ± 14.25	64.43 ± 18.25	0.997
SBPSD	9.35 (6.16, 12.70)	8.37 (6.56, 14.32)	0.739
DBPSD	8.475 (6.28, 13.05)	9.59 (6, 14.39)	0.986
SBPV (%)	0.085 (0.05, 0.11)	0.07 (0.05, 0.11)	0.359
DBPV (%)	0.11 (0.08, 0.18)	0.11 (0.07, 0.19)	0.977
FVC (L)	3.43 (2.83, 4.05)	3.32 (2.62, 3.86)	0.424
FEV1 (L)	2.52 (2.12,3.09)	2.41 (2.03, 2.86)	0.411
MVV (L/min)	75.55 (56.98, 89.88)	72.9(57.8, 83.3)	0.458
6MWT (m)	523.5 (482.75, 545)	534 (492, 598)	0.293
ET-1 (nmol/L)	0.21 ± 0.05	0.24 ± 0.05	0.011*
NO (nmol/L)	0.33 (0.27,0.36)	0.225 (0.20, 0.27)	0.000**
GS	0.5 (0.5, 1.375)	10 (6, 23)	0.000**

*P<0.05,**P<0.01

### Relationship between GS and study variable

The associations between GS and the study variable were examined using Spearman’s or Pearson’s correlation analysis. We found GS to be positively correlated with ET-1 (P < 0.01) (Table 2). Additionally, GS was negatively correlated with NO (P < 0.01), T4 (P < 0.01), and TSH (P < 0.01). Furthermore, no significant correlation was observed between GS and another study variable (Table 2).

**Table 2:** Relationship between GS and study variable (N = 71)

Variable (instrument)	Value	Correlation coefficient (r)
GS**	3.5 (5, 10)	—
ET-1 (nmol/L)**	0.23 ± 0.05	0.361
h (bpm)*	150.62 ± 12.42	0.274
Glu (mmol/L)**	5.24(4.79,7.26)	0.319
NO (nmol/L)*	0.27 (0.23, 0.33)	-0.596
T4 (nmol/L)*	101.2 (90.6, 123)	-0.245

*P<0.05,**P<0.01

### GS GS by a multiple linear regression analysis

Multiple linear regression analysis was performed to assess the association between GS and relevant study variables, including serum ET-1, NO, T4, and TSH levels (model R^2^ = 0.160) (Table 3). The positive correlation between ET-1 and GS persisted (β = 0.371, P = 0.002). The result showed that serum ET-1 levels had the most significant effect on GS scores, indicating that serum ET-1 levels may be a risk factor for CCS progression as measured by CCS severity.

**Table 3:** Model for GS by a multiple linear regression (N = 71)

	Unstandardized coefficient		Standardization coefficient	t	P value
	B	Standard error	Beta		
Constant	-18.027	15.364		-1.173	0.245
ET-1 (nmol/L)**	86.858	31.258	0.327	2.779	0.007
NO (nmol/L)	-26.675	21.719	-0.149	-1.228	0.224
Glu (mmol/L)	0.065	0.435	0.017	0.150	0.882
T4 (nmol/L)	-0.035	0.060	-0.066	-0.593	0.555
h (bpm)	0.199	0.113	0.199	1.756	0.084

Dependent variable: GS, R-squared 0.242, Adjusted R-squared 0.181, **P<0.01.

## Discussion

We found significant differences between the two groups in terms of ET-1 and NO levels. The high GS group had higher ET-1 expression levels and relatively lower NO expression than the low GS group. Moreover, GS levels were positively correlated with ET-1 and negatively correlated with NO. However, further analysis revealed no association of GS with cardiopulmonary function, suggesting that collateral circulation is often associated with stable CHD.

GS is an easy-to-use and powerful tool for assessing coronary arteries stenosis severity and complexity [19]. GS is used to assess stenosis severity. The etiopathogenesis of coronary stenosis is mainly atherosclerosis [22]. The lesion starts from the intima damage, and several types of lesions, including accumulation of lipids and complex sugars, fibrous tissue hyperplasia, and calcification, and gradual degeneration of the middle layer of the artery, occur. In the past few decades, GS has found widespread application to assess the degree of CCS severity. An observational study reported that GS values can predict periprocedural myocardial infarction [17]. Moreover, another study found that GS is an independent predictor of long-term adverse outcomes in patients with CCS who underwent PCI [19].

ET-1 and NO are two essential biomolecules that play a critical role in CCS development. Endothelial cells when stimulated by factors such as epinephrine, thromboxane, vasopressin, angiotensin, insulin, and cytokines synthesize and release ET-1 [9]. ET-1 is an active polypeptide with potent vasoconstriction and has a crucial physiological role in regulating normal cardiovascular function, especially vascular endothelial cells, smooth muscle cells, and cardiomyocyte functions. ET-1 has been demonstrated to play a role in endothelial dysfunction and inflammation, both of which areactively involved in the pathophysiology of the onsetand progression of coronary artery disease, from the formation of atherosclerotic plaque to the development of acute coronary syndrome and heart failure following myocardial infarction. [23] ET-1 has been assessed as a predictor of and prognostic marker in CCS, myocardial infarction, and heart failure. The association of ET-1 levels with mortality in the general population has also been explored. The results have shown that ET-1 is a valuable marker for risk stratification in this setting, and elevated high levels of ET-1 are independent predictors of long-term all-cause mortality, major adverse events, and cardiovascular mortality [24]. In our study, the correlation analysis between ET-1 and other parameters showed that ET-1 is negatively correlated with NO.

Contrarily, ET-1 is positively correlated with GS. GS represents the coronary artery lesion assessment based on CAG. The fact that GS is positively correlated with ET-1, while ET-1 can reflect the degree of vascular endothelial damage, suggested that clinically, GS and ET-1 can be used to independently and indirectly evaluate the degree of systemic vascular diffuse obstruction or coronary artery conditions.

When arginine is catalyzed by NO synthase, NO is produced [7], and it mediates smooth muscle relaxation, neurotransmission, and inflammation regulation in many organ systems and pathophysiological conditions. Reduced plasma NO levels are reportedly significant risk factors for CCS development and severity [12], and an increase in NO bioactivity can treat CCS [6]. In the present study, GS was negatively correlated with NO. Consequently, low NO levels are considered another significant risk factor for CCS. Overall, the imbalance between ET-1 and NO levels may disrupt vascular function and contribute to atherosclerosis development, thereby increasing CCS risk. Therefore, regulating ET-1 and NO levels may be a promising strategy for CCS prevention and treatment. Of note, the mechanism of action of ET-1 and NO is extremely complex and influenced by multiple physiological and pathological factors. Therefore, further studies are needed to confirm this specific relationship.

Studies have shown that patients with hyperthyroidism may have higher GS. Hyperthyroidism may lead to pathophysiological processes, such as vascular endothelial cell damage, platelet aggregation, and thrombosis, which may increase the incidence and severity of coronary artery disease. Furthermore, hypothyroidism is also associated with coronary artery disease. One study found that elevated TSH levels were associated with an increased risk of coronary heart disease and thyroid hormone supplementation may help improve coronary heart disease symptoms and prognosis. In our study, thyroid function abnormalities were not statistically different between the two groups, probably due to the small sample size. However, correlation analysis showed that GS scores were negatively correlated with TSH and T4. Therefore, abnormal thyroid function may impact the development and severity of coronary heart disease; hence, there may be a relationship between T4 and TSH levels and GS. Nevertheless, more studies are needed to confirm the exact relationship.

Additionally, we also found no significant correlation between GS and various cardiopulmonary function indicators. The above results further confirmed the absence of a direct relationship between the degree of diffuse coronary vascular lesions and cardiopulmonary function. In long-term coronary artery lesions, coronary artery stenosis can cause perfusion pressure imbalance and cause the opening of the existing collateral circulation. This “auto bypass” not only exists in chronic occlusive lesions but also plays an essential protective role in STEMI [25].

## Limitations

First, this was a single-center study on patients admitted for CCS and no healthy individuals were included as controls. Second, the study had a small sample size. Third, follow-up observation was not performed. Future studies with long-term follow-ups are needed to evaluate the relationship between GS and serum markers and their guiding significance for clinical application.

## Conclusion

The present data suggest a significant relationship between ET-1, NO, and GS, with increased ET-1 levels and decreased NO levels correlating with an increased GS. Their combined use may be an essential predictor of CCS disease severity.

## Data Availability

The datasets used during the current study are available from the corresponding author on reasonable request.
